# Elevated titanium levels in Iraqi children with neurodevelopmental disorders echo findings in occupation soldiers

**DOI:** 10.1007/s10661-014-4127-5

**Published:** 2014-12-03

**Authors:** M. Savabieasfahani, S. Alaani, M. Tafash, S. Dastgiri, M. Al-Sabbak

**Affiliations:** 1 P.O. Box 7038, Ann Arbor, MI 48107 USA; 2 Fallujah General Hospital, Althubbadh District, Fallujah, 00964 Iraq; 3 Medical College, Al-Anbar University, Fallujah, 00964 Iraq; 4 Department of Community and Family Medicine, School of Medicine, Tabriz University of Medical Sciences, Tabriz, 5166615739 Iran; 5 Department of Obstetrics and Gynecology, Basra Maternity Hospital, Basra Medical School, P.O. Box 1633, Basra, Iraq

**Keywords:** War pollution, Neurodevelopmental disorders, Hawija, Fallujah, Titanium, Magnesium

## Abstract

Anthropogenic release of pollutants into the environment is especially harmful to growing fetuses and young children. These populations are at an increased risk of damage because exposure to pollutants during critical periods of development can cause many impairments. Children’s exposure to mixtures of metals could be responsible for the rising numbers of neurological disorders surfacing in Iraqi children. Titanium (Ti) and magnesium (Mg) are heavily used in war industries. Exposure to Ti and Mg has been linked to the dust in occupation soldiers’ lungs. Hair samples of children in Hawija, Iraq (*n* = 13) contained significantly higher levels of Ti compared to Iranian children (*n* = 13) living near the Iraqi border (2080 ± 940 vs 707 ± 421 μg/kg, *p* < 0.0001). Magnesium was 1.7 times higher in Hawija children compared to Iranian children (115,763 ± 118,155 vs 67,650 ± 46,729 μg/kg). In samples from Hawija, Ti was 1.3 times higher in children with neurodevelopmental disorders (2198 ± 1108 vs 1942 ± 779 μg/kg), and Mg was 1.9 times higher in children without neurodevelopmental disorders (155,618 ± 140,791 vs 81,602 ± 91,940 μg/kg). Lead, arsenic, and cadmium in Hawija children with neurodevelopmental disorders (*n* = 6) were 2.5, 2.2, and 1.37 times higher compared to non-disabled children (*n* = 7). To get a clear understanding of the current status of neurodevelopmental disorders in Iraqi children and to determine the magnitude of this suspected global health issue, registries should be set up to compile and aggregate data from hospitals, clinics, and health centers across the country. Functional registries can develop collaborations with researchers toward finding causes of these disorders in Iraqi children and toward preventing them.

## Introduction

Global public health is harmed by the anthropogenic release of pollutants into the environment. Mining, waste incineration, hazardous waste sites, and war have been shown to release harmful toxicants into otherwise healthy environments, putting populations residing nearby at risk for adverse health impacts (Shields et al. 1992; Liao et al. 2010; Cordier et al. 2004; Wright et al. 2006; Al-Sabbak et al. 2012). The most vulnerable populations (i.e., elderly, pregnant mothers, growing fetuses, and young children) are most severely affected by the environmental release of toxicants.

It is now widely accepted that environmental pollutants, including metals, can disrupt neurodevelopmental processes during critical periods of development, resulting in effects on sensory, motor, and cognitive function. We now know that developmental exposure to chemicals can have adverse effects on the structure and/or function of the nervous system and can harm neurodevelopmental processes (Schardein and Keller 1989; Jurewicz et al. 2013). Among metals, lead and mercury are recognized causes of neurodevelopmental disorders as well as subclinical brain dysfunction. Prenatal exposures to these metals during in utero development can cause brain damage in the developing fetus. Fetal brain damage can result from metal exposure levels which are much lower than those affecting the adult brain and its normal function. Increasing evidence suggests that chemicals can also be the cause of neurodevelopmental damage in the unborn.

The small city of Hawija is located approximately 175 km north of the capital, Baghdad. In 2004, a school was taken over and converted into an American military base, Operating Base McHenry. Overall, a total of 200 military camps, 141 forward operating bases, and 69 combat outposts have operated in Iraq since the 2003 invasion. Multiple waste types, generated by these military installations, have been disposed of in massive open-air burn-pits across Iraq (Kennedy 2008; Jacobs 2013; Woodall et al. 2012). Another major concern in Iraq has been exposure to uranium. A 2007 publication of the United Nations Environment Program (UNEP) estimated that 1000 to 2000 metric tons of depleted uranium were fired during the 2003 war in Iraq.

It has been reported that, under the Logistics Civilian Augmentation Program (LOGCAP), open-air burn-pits, as wide as 10 acres, continuously burned waste on US military bases throughout Iraq until 2010. After considerable health complaints from the US military personnel, the US Congress voted to prohibit the burn-pits, with an amendment to the National Defense Authorization Act. Subsequently, federal law required the establishment of a registry for eligible individuals who may have been exposed to toxic airborne chemicals and fumes caused by those open burn-pits (Kime 2013; Hansia 2014). Jet fuel was commonly utilized to burn and dispose of plastics, batteries, appliances, medicine, dead animals, and even human body parts in these open burn-pits. A recent report has suggested that styrofoam (i.e., styrene), electronics, rubber tires, explosives, and asbestos insulations have also been disposed of in these open-air burn-pits (Kennedy 2008).

Styrene is a known neurotoxicant. In humans, chronic exposure to styrene has been linked to effects on the central nervous system. In addition, an increased frequency of spontaneous abortions and a decreased frequency of births have been reported in a study on the reproductive effects of styrene in humans (ATSDR, Toxicological Profile for Styrene; HSDB, online database). Cadmium, copper, and lead are created in abundance when burning electronic waste and plastics (Brigden et al. 2005; Nnorom and Osibanjo 2009). Similarly, because toxic heavy metals are an integral part of rubber, explosives, and batteries, emanations laden with heavy metals can result from burning them (Ahamd et al. 2009; Bushuyev et al. 2012; Cameron et al. 2011). Increasingly, emissions from the burning of such compounds have been scrutinized as a significant global source of harmful pollutants.

During the past decade, hundreds of American soldiers, who temporarily lived on various US military bases in Iraq and Afghanistan where open burn-pits were heavily used to dispose of waste, have reported medical problems as a result of exposure to those burn-pits. A few studies have examined this problem (Conlin et al. 2012; Smith et al. 2012; King et al. 2011). Additionally, recent investigations have linked titanium (Ti) and magnesium (Mg) to the dust found in Iraq and Afghanistan veterans’ lungs (Szema et al. 2014). Both metals are heavily used in the war industry and in the manufacture of weaponry. While soldiers’ exposure to toxic compounds is transient and will discontinue after they leave the polluted environment, the local populations’ exposure to toxic pollutants remains uninterrupted. We therefore expect Hawija residents to be chronically exposed to a persistent cocktail of toxic metals.

Parallel with this environmental condition, doctors and health professionals in Hawija have been witnessing increasing numbers of children with neurological disorders. Increases in birth defects and adverse reproductive outcomes have been linked to public contamination with lead and mercury in two other Iraqi cities, Fallujah and Basra, where numerous US military installations have also been operating since 2003.

In this setting, we hypothesized that the hair metal content of Hawija children with neurodevelopmental disorders would be higher than that of non-disabled children living in the same town. We also expected to see a continuum of decreasing severity of neurodevelopmental disorders (Hawija < Fallujah and Basra) as we move away from areas with large aggregates of military bases and with a history of heavy urban military bombardments.

## Materials and methods

### Study area

**Fig. 1:**
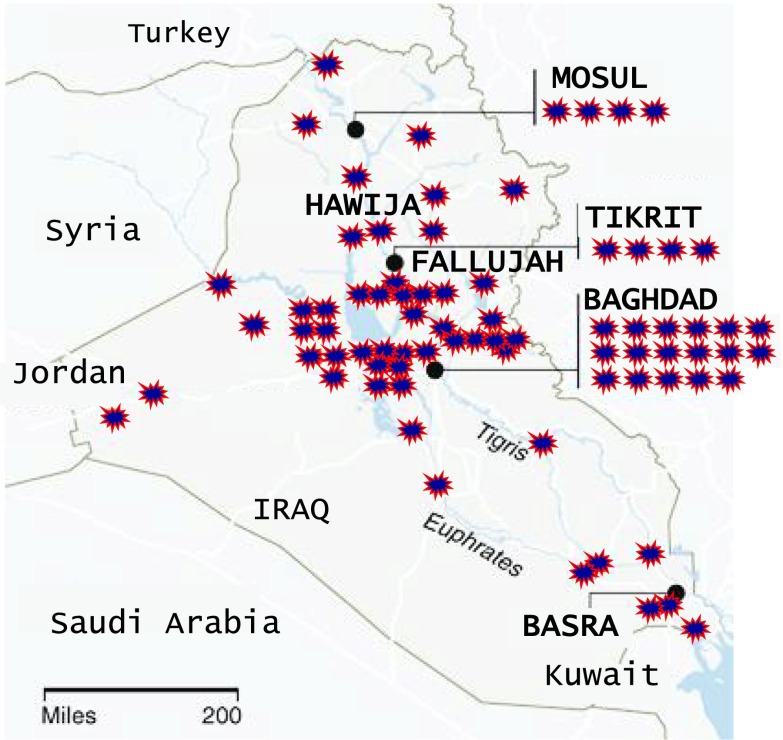
Map of major US military installations in Iraq since 2003. Hawija is approximately 175 km north of the Capital, Baghdad

Hawija is a city of 40,000 people located 175 km north of Baghdad (Fig. 1). The latitude (34.00371) and the longitude of Hawija (44.39538) have been reported using a global positioning system (GPS). The prevailing climate in Hawija is known as a local steppe climate. Throughout the year, there is little rainfall. The Köppen-Geiger climate classification considers Hawija a hot semi-arid climate, with generally rainless summers and wetter winters. Temperatures in Hawija often reach 56 °C in the long and dry summer. A National Environmental Strategy for Iraq publication which was released in 2012 suggests that desertification has had a negative impact on the environment and has directly affected the life of the population by increasing the rates and frequencies of sand and dust storms in our study area to unprecedented levels.

### Questionnaires, participant consent, and sample collection

In September 2013, local health workers and medical staff in Hawija sought participants for a metal biomonitoring study of the city. Health workers recruited seven mothers and two of their children into this study. Each mother enrolled two siblings into the study, one non-disabled child (*n* = 6) and another child with a disability (*n* = 7). Using a 48-item questionnaire, mothers were interviewed and signed a consent form permitting the team to use all information for research purposes only. Mothers also consented to the collection of hair samples from their children for metal analysis. Utilized questionnaire examined the reproductive history of the mother, residence history for the family, health and disease during pregnancy, drug use during pregnancy, smoking and alcohol use, source of water for the family, and exposure to potential war contaminants. Simultaneously, a uranium exposure and contamination self-assessment questionnaire with checklists was also completed by each participating mother (Table 1). This tool was designed to determine the movement of the individual into and out of polluted environments, the duration of such events, and potential physical manifestations of exposures (i.e., nose bleeds, skin irritation or stinging sensations, coughs, etc.). At the same time, children’s hair (approximately 0.5 g) was collected, with scissors, from the nape of the head and placed in clean paper envelopes, then sealed and transported to the laboratory.

**Table 1:** Results of uranium exposure or contamination self-assessment questionnaire, responses of Hawija mothers who participated in the current study in September 2013

Uranium exposure and contamination self-assessment questionnaire							
	Mother’s responses						
	8b	3b	10a	1b	5b	2a	6a
Was your residence ever bombed?	Yes	Yes	Yes	Yes	Yes	Yes	Yes
Were you in your house when it was bombed?	Yes	Yes	Yes	No	No	Yes	Yes
Do you live near a military installation?	Yes	No	No	Yes	No	No	Yes
During bombings did you experience any of the following?							
Nose bleed or runny nose	Yes	Yes	No	No	No	No	No
Throat, nose, or mouth irritation or stinging	Yes	Yes	Yes	No	No	Yes	Yes
Skin or eye irritation or burning	Yes	Yes	Yes	Yes	Yes	No	Yes
Dry coughs	Yes	Yes	Yes	No	Yes	Yes	Yes
Cold and flu like symptoms	Yes	Yes	Yes	Yes	Yes	Yes	Yes
After bombings did you experience any of the following?							
Unusual tiredness, fatigue, weakness	Yes	Yes	Yes	Yes	Yes	Yes	Yes
Intermittent fevers	Yes	Yes	Yes	Yes	Yes	Yes	Yes
Sweeting at night	Yes	Yes	No	No	Yes	Yes	Yes
Short-term memory loss	No	Yes	Yes	Yes	Yes	Yes	Yes
Disorientation or confusion	Yes	Yes	Yes	Yes	Yes	Yes	Yes
Depression or loss of initiative	No	No	Yes	No	Yes	Yes	Yes
Headaches	Yes	Yes	Yes	Yes	Yes	Yes	Yes
Recurring or continuous pain	Yes	Yes	Yes	Yes	Yes	Yes	Yes
Chronic cold or flu	Yes	Yes	Yes	No	No	Yes	Yes
Asthma, chronic bronchitis	Yes	No	No	No	No	Yes	Yes
Stinging sensation when urinating	No	Yes	Yes	Yes	Yes	Yes	Yes
Gastrointestinal problems	No	No	Yes	Yes	Yes	Yes	Yes

### Hair samples’ treatment, digestion, and ICP-MS analysis

The certified reference materials (NCS DC 93347 and NCS ZC 81002) were purchased from Brammer Standard Company, Inc. (Benfer Rd Houston, TX) and prepared using two different methods. One was closed microwave digestion, and another was hot block digestion. Hot block digestion was performed at a lower temperature to avoid Hg loss. Data generated from hot block and microwave digestion procedures were consistent between the two digestion methods, indicating the appropriateness of either procedure for sample digestions. Once samples were digested, digests were analyzed for multiple elements on the Agilent 7700x inductively coupled plasma-mass spectroscopy (ICP-MS). The limit of detection for the method is expressed as the mean blank signal +3× standard deviation of the blanks (at least 6 replicates) for each of the elements in micrograms per kilogram: Al < 1000; Mg < 600; Ti < 50; Cu < 90; As < 60; Cr < 40; Se < 30; V, Mn, and Pb < 20; Hg and Th < 10; Zn < 5; Cd < 4; Ni < 3; Fe, Co < 2; Mo, U < 1.

### All hair samples were hot block digested for analysis

For hot block digestion, hair was placed in clean polypropylene tubes, rinsed with deionized (DI) water once, and then rinsed with 5 ml of methanol. Samples were then washed twice with DI water and dried in an incubator for 8 h at 50 °C. Accurately weighed 0.05 g of hair, along with NCS ZC 81002 Standard Reference Material for hair, was put in a clean tube and 0.5 ml of concentrated HNO_3_, and 0.5 ml of H2O2 was added. Samples were then heated in a hot water bath for 1 h at 80 °C, and then allowed to cool. Of DI water, 9 ml was added, and tubes containing samples were then capped and shaken well before ICP-MS analysis.

For microwave digestion, samples were similarly washed and dried. Then, 0.1 g of clean and dried hair and standards were placed in a clean microwave vessel, and 2 ml of concentrated HNO_3_ and 2 ml H2O2 were added. The reaction was allowed to subside after approximately 30 min to 1 h; then, vessels were placed in a microwave for 40 min for digestion at 50 % of full power wattage. Microwaved samples were removed from the microwave and allowed to cool to room temperature. Digested samples were then transferred into clean tubes, and 6 ml of DI water was added. Samples were capped and shaken before ICP-MS analysis.

### Statistical analysis

An IBM SPSS software (version 21) was used for all analysis. A one-way analysis of variance was followed by post hoc Bonferroni multiple comparisons test to determine the source of significance. Significance level was set at *p* < 0.05.

## Results and discussion

**Fig. 2:**
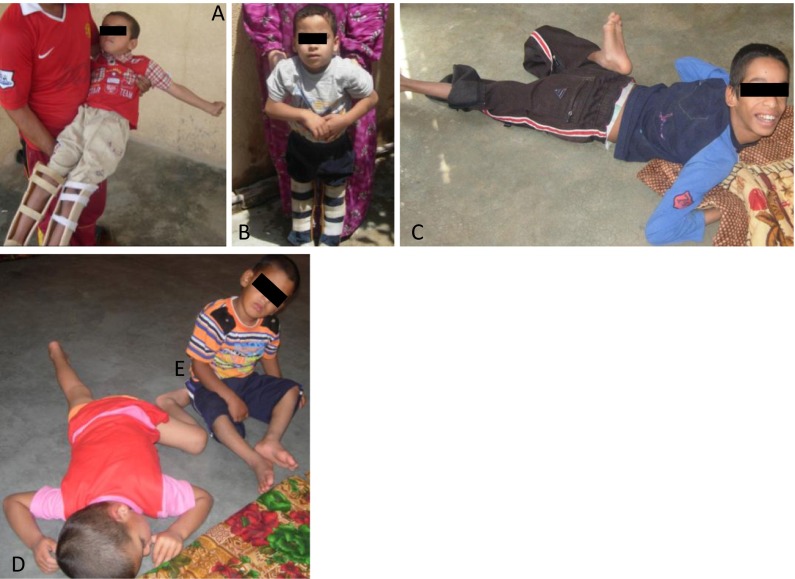
Photo of the participating children: *a* and *b* brain disorder and disfigured limbs; *c*, *d*, and *e* epileptic with general body seizures

The average age of the participating mothers (*n* = 7) was 30 ± 4.75 (range 22 to 34). Children with developmental disability (*n* = 7) were on the average 4.14 ± 2 years old (range 6 to 1). Non-disabled children (*n* = 6) were 5 ± 4.32 years old (range 12 to 1). Gestational age for children with developmental disability and non-disabled children were 36.67 ± 0.8 and 37 ± 0.7 weeks, respectively. Mothers did not smoke or drink during pregnancy, and only one mother took painkillers according to the doctor’s prescription. No one had taken antidepressants during pregnancy. A total of six miscarriages and four stillbirths were reported by the mothers. Two miscarriages had occurred in 1992 and one in 1998. The remaining miscarriages had occurred after 2003. Children with neurodevelopmental problems in this study were diagnosed by licensed local physicians suffering from brain damage, epilepsy, continuous body seizures, missing fingers and toes, or disfigured limbs (Fig. 2.)

Table 2 reports metal levels (mean ± STDEV in μg/kg) measured by ICP-MS. We found that the levels of toxic metals, including lead (Pb), arsenic (As), and cadmium (Cd) in children with neurodevelopmental disorders (*n* = 6), were 2.5, 2.2, and 1.37 times higher compared to that of non-disabled children (*n* = 7). Mercury hair content was 792 ± 1207 μg/kg in the non-disabled children and 698 ± 1190 μg/kg in the disabled children. Reported differences were not statistically significant (*p* > 0.05). Children’s uranium exposure appeared to be low in both non-disabled and disabled Hawija children.

**Table 2:** List of metals in children’s hair from Hawija, and previously reported values from Fallujah analyzed by ICP-MS

Hair metal	Mean ± STDEV (range) μg/kg Hawija children		Mean ± STDEV μg/kg Fallujah children	
	Normal (*n* = 6)	With neurodevelopmental disorder (*n* = 7)	Normal (*n* = 11)	With birth defects (*n* = 31)
Cr	436.3 ± 417 (91–1158)	282.7 ± 64.2 (184–343)	748 ± 412	393 ± 335
As*	83 (>20–83)	180 ± 94 (112–319)	148 ± 70	145 ± 111
Cd*	83 ± 68 (7–164)	114 ± 77 (16–208)	72 ± 69	221 ± 786
Hg	792 ± 1207 (43–3191)	698 ± 1190 (39–3250)	1414 ± 3854	8282 ± 25,844
Pb*	3714 ± 2216 (1149–4976)	9181 ± 9752 (370–26,245)	11,277 ± 27,781	34,022 ± 128,815
Mn*	2415 ± 1718 (304–5125)	2915 ± 2761 (250–4049)		
Al*	29,883 ± 18,377 (13,531–57,022)	30,475 ± 16,973 (10,004–56,846)		
V*	275 ± 75 (<20–356)	422 ± 214 (<20–624)		
Fe*	31,566 ± 17,345 (12,684–56,776)	35,545 ± 17,053 (16,639–65,808)		
Co*	49 ± 27 (9–83)	69 ± 60 (180–12)	301 ± 210	89 ± 53
Ni*	400 ± 220 (108–687)	427 ± 262 (126–925)		
Cu	43,732 ± 76,504 (5634–199,722)	13,803 ± 6134 (8213–23,463)		
Zi*	173,738 ± 86,459 (94,975–321,590)	183,400 ± 94,622 (85,445–363,804)		
Se	601 ± 55 (518–676)	512 ± 189 (202–734)		
Mo*	56 ± 28 (19–94)	72 ± 53 (29–180)		
U*	16 ± 17 (3–50)	19 ± 13 (4–46)	61 ± 41	36 ± 41

Values are mean ± standard deviation

*Asterisk* larger numerical values in Hawija children with neurodevelopmental disorders compared to normal children from the same city, but no statistical differences (*p* > 0.05)

Mothers’ responses to the uranium exposure and contamination self-assessment questionnaire are provided in Table 1. All of the participants’ homes had been bombed at least once, and four of their neighbors’ houses had also been bombed. Three of the residences of participants had been the target of white phosphorous attacks. Three out of the seven mothers said they lived near the military base in town. Cold and flu-like symptoms; unusual tiredness, fatigue, or weakness; intermittent fevers; disorientation or confusion; headaches; and recurring or continuous pain (in legs and back) were most frequently recalled by the mothers during and after bombardment. Nose bleed or runny nose, and asthma and chronic bronchitis were least reported. Half of the participants reported depression or loss of initiative following bombardment. Our data suggests that participants had been exposed to varying degrees of pollution created by bombing or by air pollution as a result of living near a military base with open burn-pits. Children’s uranium exposure appeared to be low in both non-disabled and disabled Hawija children (Table 2).

**Fig. 3:**
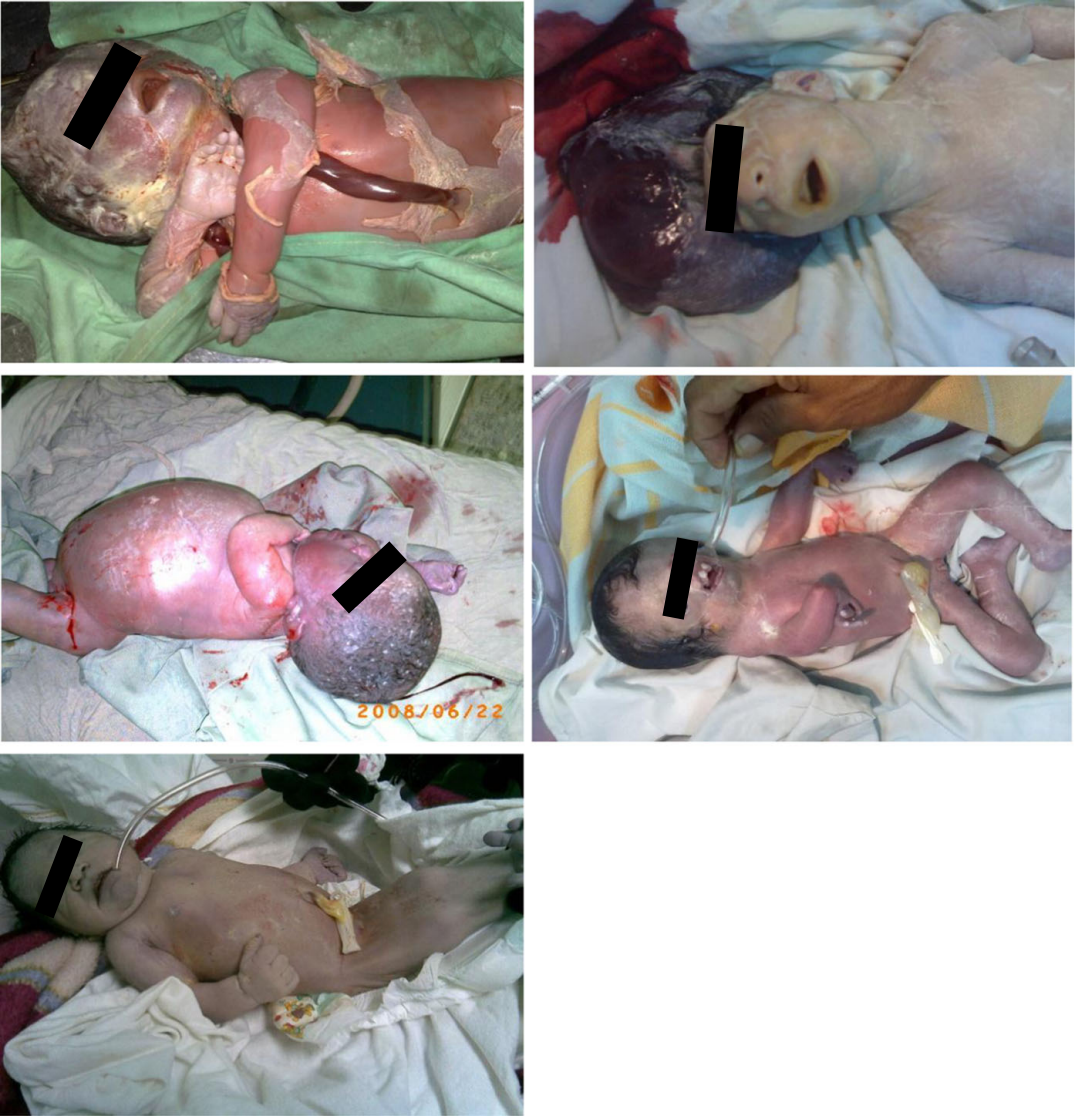
Selected photos of children born in Fallujah between 2008 and 2010, showing severe neurodevelopmental disorders, in the form of multiple birth defects

**Fig. 4:**
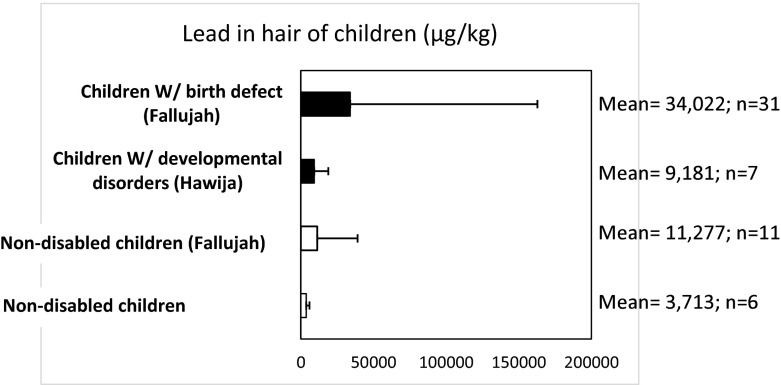
A comparison of lead in children’s hair samples from Hawija and Fallujah (μg/kg)

Previous reports of hair metal content in children with birth defects from Fallujah, Iraq (Al-Sabbak et al. 2012; Alaani et al. 2011) show 3.7 times higher lead than that of the levels we report in Hawija children with neurodevelopmental disorders. Mercury was 12-fold higher in Fallujah children with birth defects compared to Hawija children with neurodevelopmental disorders. Limited numbers of observations in some groups, combined with high variability in metal levels within groups, make data interpretation difficult. However, a trend is detectible (Figs. 3 and 4). In Iraqi children, born with neurological disorders, the severity of neurodevelopmental effects is more pronounced as lead levels increase. The city of Fallujah is surrounded by military bases and has been the target of more bombings compared to that of Hawija, where bombings have been less frequent and the numbers of military bases are also fewer (Fig. 1). It can therefore be argued that children of Fallujah—who have a higher likelihood of exposure to war-related pollutants—exhibit more severe neurodevelopmental conditions than that of children of Hawija.

### Titanium and magnesium are elements of the war industry

Titanium and Mg are integral to the war industry, and both elements are key to the manufacture of weaponry. Titanium has been widely used in the US military since the 1960s. It is favored because it weighs less than other metals and it does not rust. It is estimated that about 55 % of the Ti manufactured in North America is utilized in the military and aerospace industries. Titanium is used extensively in US weapons systems, machine guns, ground vehicles, combat vehicles, weapons platforms, tanks, armored personnel carriers, pressure vessels in ballistic missiles, and in Blackhawk and McDonnell Douglas Apache helicopters (Titanium Structures for Army Systems, SM2 KN4-1).

Similarly, Mg has ballistic and structural applications in the military, and its use has been growing since the Second World War. Magnesium is widely used in the making of tanks, artillery, armored vehicles, and other military equipment. Magnesium is utilized by the military as a major incendiary agent. It has been used in cluster bombs for its capacity to burn persistently for an appreciable length of time with a very high temperature and cannot be easily extinguished (Jones et al. 2012).

Exposure to Ti and Mg has been linked to dust found in the lung tissue of US occupation soldiers (Szema et al. 2014). Hair samples of Hawija children (*n* = 13) contained three times more Ti (2080 ± 940 μg/kg) than that of hair samples of Iranian children (*n* = 13) who live in Khoram Shahr near the Iraqi border (707 ± 421 μg/kg; unpublished forthcoming data, *p* < 0.0001). Magnesium was 1.7 times higher in Hawija children than in Iranian children (115,763 ± 118,155 vs 67,650 ± 46,729 μg/kg). Ti levels are rarely reported in children’s hair; however, Table 3 contains a current review of the literature on Ti and Mg levels in children’s hair. Titanium in hair samples from Hawija have the highest levels of this metal ever reported in children’s hair globally.

**Table 3:** A review of literature on Ti and Mg levels in children’s hair

Reference	Year	Country	*N* Children	Instrument	Titanium (μg/kg)	Iron (μg/kg)	Magnesium (μg/kg)
Blaurock-Busch et al.	2011	Egypt	25	ICP-MS	560	11,000	70,000
Peña-Fernández et al.	2014	Spain	117	ICP-AES	900	–	–
Raposo et al.	2014	Spain	112	ICP-MS	1300	17,400	61,000
Senofonte et al.	2000	Italy	396	ICP-AES	790	19,000	28,000
Forthcoming, unpublished		Khoram Shahr, Iran	13	ICP-MS	707	22,969	67,650
This study	Present	Hawija, Iraq	13	ICP-MS	2080*	33,708	115,763
Park et al.	2007	Korea	655	ICP-MS	–	12,290	12,620
Al-Farsi et al.	2013	Oman	27	ICP-MS	–	46,000	18,00
Vanaelst et al.	2013	Belgium	164	ICP-MS	–	10,000	34,000

*ICP-AES* inductively coupled plasma atomic emission spectroscopy, *ICP-MS* inductively coupled plasma-mass spectroscopy

*p < 0.0001, one-tailed *t* test

In samples from Hawija, Ti was 1.3 times higher in children with neurodevelopmental disorders (2198 ± 1108 μg/kg) than that in children without neurodevelopmental disorders (1942 ± 779 μg/kg). Mg was 1.9 times higher in children without neurodevelopmental disorders (155,618 ± 140,791 μg/kg) than that in those with the disorder (81,602 ± 91,940 μg/kg). Interestingly, Mg has been shown to protect against brain damage by diminishing neuronal apoptosis (Turkyilmaz et al. 2002). Moreover, a review of the literature has found associations between Mg treatment and significantly reduced risk of infant mortality and cerebral palsy (a neurodevelopmental disorder of major concern). Antenatal treatment with Mg during premature deliveries has been suggested to have health benefits for the infant (Wolf et al. 2012). Our findings corroborate with the available literature on the beneficial and protective effects of Mg on brain development. Hawija children with higher Mg levels appear to have been protected against neurodevelopmental damage.

In Iraq, an estimated 1000 to 2000 metric tons of depleted uranium was fired during the 2003 US invasion of that country (UNEP 2007, Annual Report). The explosion of depleted uranium bombs can develop temperatures that exceed 3000 °C (annual report, 1978). The magnitude of this combustion can vaporize everything found on battlegrounds, including Ti and Mg containing material. As the vaporized materials cool, nanoparticles are created and are scattered in the environment. Inhalation or ingestion of these mainly metallic particles can cause pathologies in humans (Gatti and Handbook 2005; Nemmar et al. 2002).

Recent laboratory studies have linked in utero titanium nanoparticle exposures to brain cell necrosis, hippocampal cell apoptosis, and neurotoxic effects in offspring (Ze et al. 2014; Mohammadipour et al. 2014), implying potential for brain damage in the exposed offspring. It has been suggested that the interaction of titanium dioxide nanoparticles with other chemicals increases toxicity, heightens damage to cells, and aggravates pathologies (Liu et al. 2014).

The Iraqi public, including the most vulnerable populations of pregnant women and children, may have been cumulatively exposed to metals including Ti and Mg nanoparticles. Such exposures can cause various impairments.

Hair metal studies which relate war-contaminant exposure to neurodevelopmental disorders warrant more research to clarify the effects of war-related pollutants on Iraqi children’s health. Registries need to be established to compile and aggregate data from hospitals, clinics, and health centers across the country, including Hawija, Fallujah, and Basra. Data from these registries can then be used to guide researchers in developing large-scale epidemiological studies to determine risk factors, to develop intervention strategies, and to implement plans to protect mother-child health in Iraq. The registries will be instrumental in understanding the impact of birth defects in Iraq.

### Exposure to mixtures of chemicals and children’s health

In developing fetuses and young children, windows of heightened sensitivity to toxic exposures have long been identified and acknowledged (Goldman 1995). Concurrently, several studies indicate that metals interact to cause health effects which differ from those caused by exposure to individual metals alone. Current literature supports the assertion that exposure to mixtures of metals may have additive or synergistic effects that can alter toxicity, especially in developing children (Claus Henn et al. 2014; Marques et al. 2014). Metals are of particular concern to children’s health, because of the relatively high probability of exposure and the ability of metals to individually cause adverse developmental and neurological effects. Interactive effects of early-life lead and manganese exposures on cognition and neurodevelopmental effects have been reported (Claus Henn et al. 2012; Lin et al. 2013). Additionally, a large cohort study has shown interactions between lead and cadmium, with effects on reproductive hormone levels and neurodevelopment (Kim et al. 2013).

Hawija children with neurodevelopmental disorders were exposed to high levels of arsenic. A recent review of the literature offers clear evidence that arsenic exposure can lead to neurodevelopmental problems in children (Parvez et al. 2011). Furthermore, a recent study found significant associations between children’s neuropsychological function and hair manganese and arsenic (Rodríguez-Barranco et al. 2013). Thus, metal mixture toxicity is a suspect in the spectrum of neurodevelopmental disorders we observe in Iraqi children living near and around areas contaminated with war-related pollutants.

The small number of recruitable participants for this study has been limiting and can be attributed to the continuous instability in the research area. That instability adds to public’s fear, insecurity, and unwillingness to participate in research projects. Nevertheless, the data we offer has been obtained from one of the most hard-to-reach geographical locations, from which no other data is currently available, adding to the strength of this research. Based on our findings, larger-scale public and environmental monitoring of the area, including monitoring of the reproductive health of the local population, is warranted.

## Conclusion

Environmental pollutants, like metals, are able to disrupt normal neurodevelopmental processes during periods of heightened sensitivity in children and growing fetuses, thereby causing adverse effects on sensory, motor, and cognitive function. Moreover, multiple metals can interact to cause health effects which are different from those caused by single-metal exposure. Current literature supports the assertion that exposure to mixtures of metals and nanoparticles that can result from high-temperature explosions of war may have additive or synergistic effects that can alter toxicity, especially in developing children. A spectrum of neurodevelopmental disorders are appearing in Iraqi cities where, for over a decade, bombing and military events have led to increased public exposures to toxic metals. To get a clear understanding of the scope of neurodevelopmental disorders in Iraqi children, registries should be set up to compile and aggregate data from hospitals, clinics, and health centers across the country. Data from these registries can then be used to guide researchers in developing large-scale epidemiological studies to determine risk factors, to develop intervention strategies, and to implement plans to protect mother-child health in Iraq.
